# Editorial for the Special Issue of 10th Anniversary of *Micromachines*

**DOI:** 10.3390/mi12010009

**Published:** 2020-12-24

**Authors:** Ai Qun Liu, Nam-Trung Nguyen, Yi Zhang

**Affiliations:** 1 School of Electrical and Electronic Engineering, Nanyang Technological University, Singapore 639798, Singapore; EAQLiu@ntu.edu.sg; 2 Queensland Micro- and Nanotechnology Centre, Griffith University, West Creek Road, Nathan, QLD 4111, Australia; nam-trung.nguyen@griffith.edu.au; 3 School of Mechanical & Aerospace Engineering, College of Engineering, Nanyang Technological University, Singapore 639798, Singapore

*Micromachines* published its inaugural issue in 2010; it has experienced a tremendous growth in both the quantity and quality of its scientific papers. *Micromachines* is a peer-reviewed open access journal on all aspects of micro/nano-scaled structures, materials, devices, and systems, as well as related micro- and nanotechnology from fundamental research to applications. Despites the growth in volume, the journal has achieved a steady growth of its impact factor. The current impact factor of 2.523 is a testament to the quality of the journal and its potential. *Micromachines* actively engages the scientific community by sponsoring scientific conference and recognizing the contribution of authors and reviewers with the “Best Paper Award”, the “Outstanding Reviewer Awards”, and the “Young Investigator Award”.

In celebration of the 10th anniversary of *Micromachines*, we have organized a 10th anniversary Special Issue to commemorate this important milestone. A total of 52 papers, including 42 original research articles, 9 review articles, and 1 brief report, are published in this Special Issue. A total of 14 out of the 52 papers in the Special Issue are selected as feature papers [1,2,3,4,5,6,7,8,9,10,11,12,13,14].

Among the feature papers, quite a few report novel platforms for biomedical applications. Antonio De Grazia et al. investigated the bacterial fouling of ureteral stents using a microfluidic model and discovered the presence of hydrodynamic cavities in the vicinity of a ureteric occlusion [1]. Their findings would lead to novel solutions against bacterial fouling by reducing the extent of cavity flow. Valentina Palacio-Castañeda et al. developed a tumor-on-a-chip model to investigate the metabolic switching of tumor cells under hypoxic conditions [2]. They discovered that a rapid metabolic switch of tumor cells under hypoxic conditions led to increased glycolysis. Davood Saeidi et al. investigated cell-particle secondary acoustic radiation forces in a plain ultrasonic standing wave field inside a microfluidic channel and discovered that the secondary acoustic force acting on biological cells could dominate the primary acoustic radiation force under certain conditions, which could potentially lead to new microscale acoustofluidic methods [3]. Mehdi Tahernia et al. developed an interesting paper-supported 3D culture platform for the high-throughput monitoring of *C. elegans* [4]. The paper substrate trapped *C. elegans* in the porous and aquatic paper matrix, and worms could grow until their body reached a size comparable to the paper pores. The development, fertility, and dynamic behavior of *C. elegans* in this paper-supported 3D culture platform outperformed those of the standard 2D cultivation technique. Lena Gorgannezhad et al. reported a microfluidic array chip for the parallel detection of waterborne bacteria by PCR [5]. The chip design allowed reagent loading into the array in a single step utilizing capillary filling, eliminating the need for pumps, valves, and liquid handling instruments. Jong Seto developed a cell-free droplet microfluidics-based assay using inert hydrogel beads and applied this method to the detection of *Pseudomonas aeruginosa* infection [6]. This platform is easily adaptable and simply programmable to fit a variety of biological and chemical sensing applications for ease of delivery and activation in remote environments, even in conditions with very little hydration. Maxwell Rumaner et al. described a thread-based low-cost microfluidic assay for the analysis of tumor tissues ex vivo [7]. Various thread materials, such as silk, nylon, cotton, and polyester, could be used for this purpose. This user-friendly device was well-suited for drug testing in low-resource settings. Jolly Hipolito et al. examined the interaction between natural killer cells and dendritic cells on a microfluidic platform, and discovered that the natural killer–dendritic cell interactions may underlie the differential maturation of immature dendritic cells by activated natural killer cells [8]. Yuguang Liu et al. demonstrated a heterogeneous immunoassay on a digital microfluidic platform coupled with conventional channel-based microfluidics [9]. The concepts presented in this work were potentially applicable in rapid neonatal disease screening using a finger prick blood sample.

A subtractive glass 3D printing technique was reported by Peng Wang et al. [10]. The authors demonstrated the fabrication of macro-scale 3D glass objects of large heights up to ~3.8 cm with an identical lateral and longitudinal feature size of ~20 μm. The high-resolution 3D printing of macro-scale objects in glass was expected to have implications in the fields of photonics, microfluidics, and high-precision mechanics.

A few MEMS sensors are reported in this Special Issue. Chaoyong Peng et al. evaluated the performance of a dense MEMS-based seismic sensor array deployed in the Sichuan-Yunnan border region in China for earthquake early warning [11]. This MEMS-based sensor could meet the requirements of dense EEW purpose and lower the total investment of the National System for Fast Seismic Intensity Report and Earthquake Early Warning project. Javier Toledo et al. developed a piezoelectric MEMS Resonator for the detection of cigarette particles [12]. The sensor working principle was based on the resonance frequency shift caused by particles collected on the resonator surface. This principle could be extended to other sensing applications, such as the monitoring of nanoparticles in a workplace environment. Seok Kim reviewed the development of micro-LEGO for MEMS fabrication [13]. In this review paper, the process components of micro-LEGO, including transfer printing with polymer stamps, material preparation, and joining, were summarized and the recent progress of micro-LEGO within MEMS applications was discussed by investigating several example devices which are partially or fully assembled via micro-LEGO. Mariusz Radtke et al. developed a reliable method of fabricating single-crystal diamond photonic nanostructures. The method could be used to fabricate single-crystal diamond scanning probes with shallowly embedded nitrogen vacancies [14]. 

We would like to take this opportunity to thank all authors for their submission as well as all reviewers for their great effort and time. We also would like to express our gratitude to the staff at the editorial office of *Micromachines* for their kind assistance. Moving forward, *Micromachines* will continue working closely with the scientific community to publish high-quality research and keep up with scientific trends in related areas.
